# Twist1 signaling in age-dependent decline in angiogenesis and lung regeneration

**DOI:** 10.18632/aging.202875

**Published:** 2021-03-25

**Authors:** Kathryn Hendee, Tendai Hunyenyiwa, Kienna Matus, Maria Toledo, Akiko Mammoto, Tadanori Mammoto

**Affiliations:** 1 Department of Pediatrics, Medical College of Wisconsin, Milwaukee, WI 53226, USA; 2 Department of Cell Biology, Neurobiology and Anatomy, Medical College of Wisconsin, Milwaukee, WI 53226, USA; 3 Department of Pharmacology and Toxicology, Medical College of Wisconsin, Milwaukee, WI 53226, USA

**Keywords:** aging, angiogenesis, lung, Twist1, PGC1α

## Abstract

Angiogenesis – the formation of new blood capillaries- is impaired in aging animals and contributes to the pathogenesis of age-related diseases. A transcription factor, Twist1, contributes to the pathogenesis of age- and angiogenesis-related diseases such as pulmonary fibrosis and atherosclerosis. However, the mechanism by which Twist1 controls age-dependent decline in angiogenesis remains unclear. In this report, we have demonstrated that the levels of Twist1 are higher, while the expression of peroxisome proliferator-activated receptor gamma coactivator 1-alpha (PGC1α) that stimulates angiogenesis, is lower in endothelial cells (ECs) isolated from aged human adipose tissues and mouse lungs compared to those from young tissues. Knockdown of Twist1 in aged human ECs increases the levels of PGC1α and angiogenic factor receptor, vascular endothelial growth factor receptor (VEGFR2), and restores EC proliferation and migration, while inhibition of PGC1α suppresses these effects. Knockdown of Twist1 in supplemented aged ECs also restores vascular networks in the subcutaneously implanted gel, while these effects are abrogated by knockdown of PGC1α. Age-dependent inhibition of post-pneumonectomy (PNX) lung growth is suppressed in Tie2-specific Twist1 conditional knockout mouse lungs, in which VEGFR2 expression increases after PNX. These results suggest that upregulation of endothelial Twist1 mediates age-dependent decline in angiogenesis and regenerative lung growth.

## INTRODUCTION

The aging population is rapidly growing worldwide; in 2050, the population ages 60 and over is estimated to reach 2.1 billion in the world. Aging is associated with impaired organ function and increased susceptibility to various diseases, including chronic lung diseases [1–5], which are associated with life-threatening cardiopulmonary complications (e.g., pulmonary hypertension, right-sided heart failure) [6, 7]. Angiogenesis is impaired in aging animals [8–12], resulting in inhibition of organ regeneration [12]. For example, post-pneumonectomy (PNX) compensatory lung growth, which requires angiogenesis, is stimulated in young people, while it is diminished in older people [13, 14]. Inhibition of angiogenesis and attenuation of lung regeneration and repair abilities in aged people contribute to the pathogenesis of age-related lung diseases [15, 16]. Thus, we need to understand how aging disrupts angiogenesis and lung regeneration.

A transcription factor, Twist1, contributes to the age- and angiogenesis-related diseases, including pulmonary fibrosis [17, 18], diabetes [19], chronic obstructive pulmonary disease (COPD) [20], cancer [21], and atherosclerosis [22, 23]. Twist1 regulates vascular development [24] and function [25] through multiple angiogenic signaling (e.g., Tie2, platelet-derived growth factor (PDGF), VEGFR2, transforming growth factor beta receptor (TGFβR)) [18, 24–28]. Inhibition of Twist1 activity increases the expression of PGC1α that stimulates mitochondrial biogenesis [29–32] and angiogenesis [29, 33–35] in fat cells [36]. PGC1α controls age-dependent mitochondrial metabolism [31] and mediates aging-related cardiovascular diseases [29, 37–41]. The involvement of Twist1-PGC1α signaling in age-dependent inhibition of angiogenesis and how it contributes to the inhibition of lung regeneration in aged animals remains unclear.

Here we have used ECs isolated from human adipose tissue and the subcutaneous gel implantation system to examine the mechanism by which aging disrupts angiogenesis. We have then picked up lung as an organ-specific model and studied the effects of aging on lung regeneration using a PNX model. We have demonstrated that angiogenesis is impaired in aged ECs through Twist1-PGC1α signaling. Knockdown of Twist1 in aged ECs increases VEGFR2 expression and restores age-related decline in angiogenesis, while these effects are suppressed by knockdown of PGC1α. Knockdown of endothelial Twist1 also restores angiogenesis and post-PNX lung growth in aged mouse lungs. Modulation of Twist1-PGC1α signaling may be a novel intervention to rejuvenate angiogenic ability in aged adults and will lead to the development of efficient strategies for aging-associated diseases.

## RESULTS

### Twist1-PGC1α mediates age-dependent decline in VEGFR2 expression

**Figure 1:**
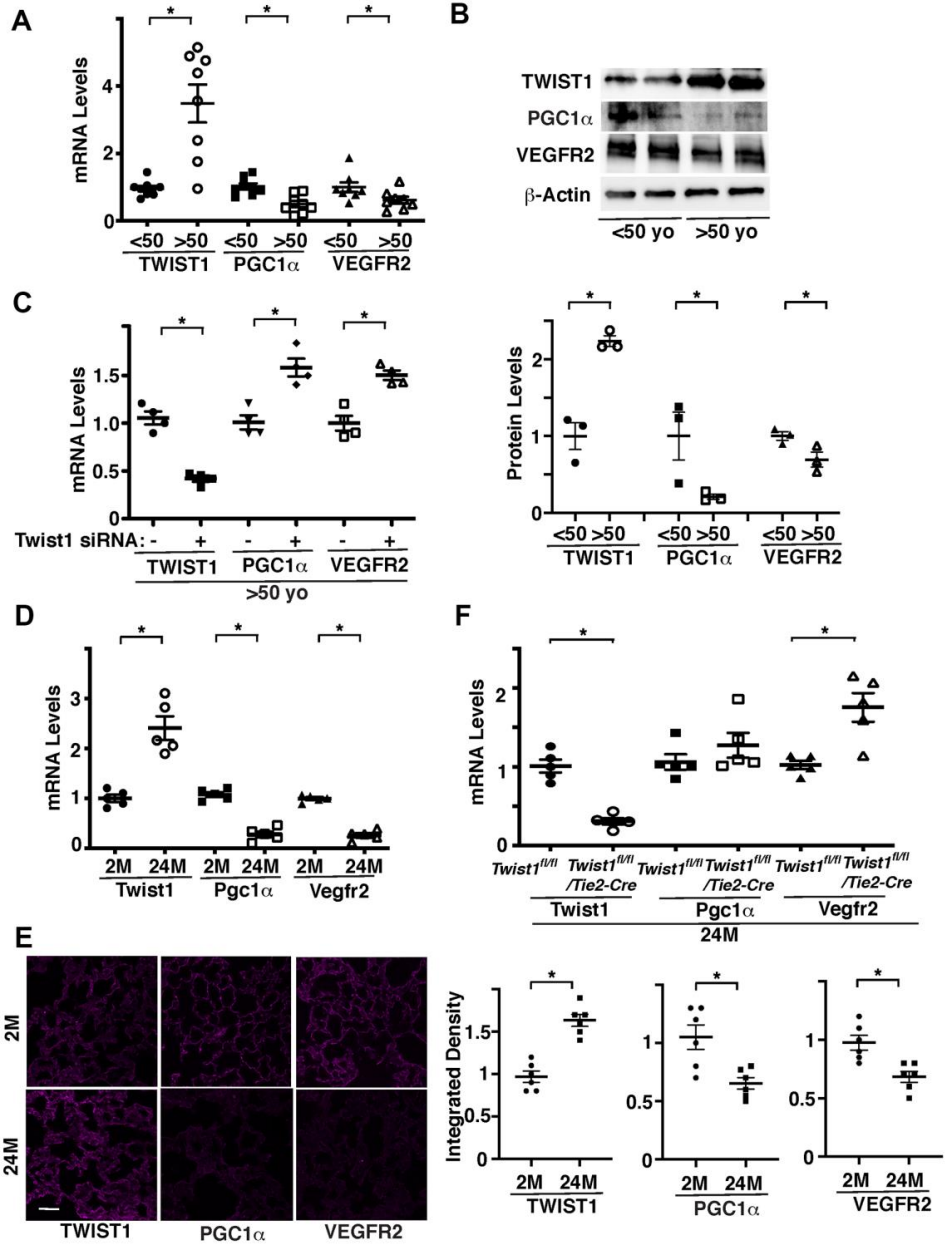
**Twist1 mediates age-dependent decline in PGC1α and VEGFR2 expression in ECs.** (**A**) Graph showing the mRNA levels of Twist1, PGC1α, and VEGFR2 in ECs isolated from young (<50 years old) vs. old (>50 years old) human adipose tissues (n=8, mean ± s.e.m., *, p<0.05). (**B**) Representative immunoblots showing Twist1, PGC1α, VEGFR2, and β-actin protein levels in young vs. aged human adipose ECs (*top*). Graph showing Twist1, PGC1α, and VEGFR2 protein levels normalized by β-actin protein levels in young vs. aged human adipose ECs (*bottom*, n=3, mean ± s.e.m., *, p<0.05). (**C**) Graph showing the mRNA levels of Twist1, PGC1α, and VEGFR2 in aged human adipose ECs treated with Twist1 siRNA or control siRNA with irrelevant sequences (n=4, mean ± s.e.m., *, p<0.05). (**D**) Graph showing the mRNA levels of Twist1, Pgc1α, and Vegfr2 in ECs isolated from 2M vs. 24M old mouse lungs (n=5, mean ± s.e.m., *, p<0.05). (**E**) IF micrographs showing Twist1, PGC1α, and VEGFR2 expression in 2M vs. 24M old mouse lungs. Scale bar, 50 μm. Graphs showing integrated density of Twist1, PGC1α, and VEGFR2 in the lung tissues (n=6, mean ± s.e.m., *, p<0.05). (**F**) Graph showing the mRNA levels of Twist1, Pgc1α, and Vegfr2 in ECs isolated from 24M old *Twist1^fl/fl^* and *Twist1^fl/fl^*/*Tie2-cre* mouse lungs (n=5, mean ± s.e.m., *, p<0.05).

Twist1 contributes to the age- and angiogenesis-related diseases, including pulmonary fibrosis [17, 18], diabetes [19], COPD [20], cancer [21], and atherosclerosis [22, 23]. We have reported that Twist1 expression is higher in the bleomycin-induced fibrotic mouse lungs [18]. Twist1 mRNA levels were 3.4- times higher in ECs isolated from discarded de-identified aged (>50 years old) human adipose tissues compared to those in the younger tissues (<50 years old) (Figure 1A). It is reported that PGC1α activates angiogenic signaling [29, 33–35] and mediates aging-related cardiovascular diseases [29, 37–41] and that inhibition of Twist1 activity in fat cells increases the activity of PGC1α [36]. Consistently, the mRNA levels of PGC1α and angiogenic factor receptor, VEGFR2 were lower in ECs isolated from aged adipose tissues by 63% and 44%, respectively, in which Twist1 expression is higher, compared to those in the younger tissues (Figure 1A). Immunoblotting results confirmed that the protein levels of Twist1 were 2.2- times higher, while the levels of PGC1α and VEGFR2 were lower by 78% and 27%, respectively, in aged human adipose ECs compared to those in the younger tissues (Figure 1B). Twist1 siRNA transfection, which decreases Twist1 mRNA levels by 62%, increased the levels of PGC1α and VEGFR2 in aged human adipose ECs by 1.5- and 1.4-times, respectively (Figure 1C).

We also confirmed the results using ECs isolated from 2 months (2M) old vs. 24M old mouse lungs. Twist1 mRNA levels were 2.4-times higher, while the mRNA levels of Pgc1α and Vegfr2 were lower by 83% and 84%, respectively, in 24M old mouse lung ECs compared to those in the 2M old mouse lung ECs (Figure 1D). Immunohistochemical analysis confirmed that the protein levels of TWIST1 were higher, while the levels of PGC1α and VEGFR2 were lower in 24M old mouse lungs compared to those in the 2M old mouse lungs (Figure 1E). The levels of Vegfr2 in ECs isolated from 24M old Tie2-specific Twist1 conditional knockout (*Twist1^fl/fl^/Tie2-cre*) mouse lungs, in which Twist1 expression is 78% decreased, were also 1.6-times higher than those in 24M old *Twist1^fl/fl^* mouse lungs (Figure 1F).

**Figure 2:**
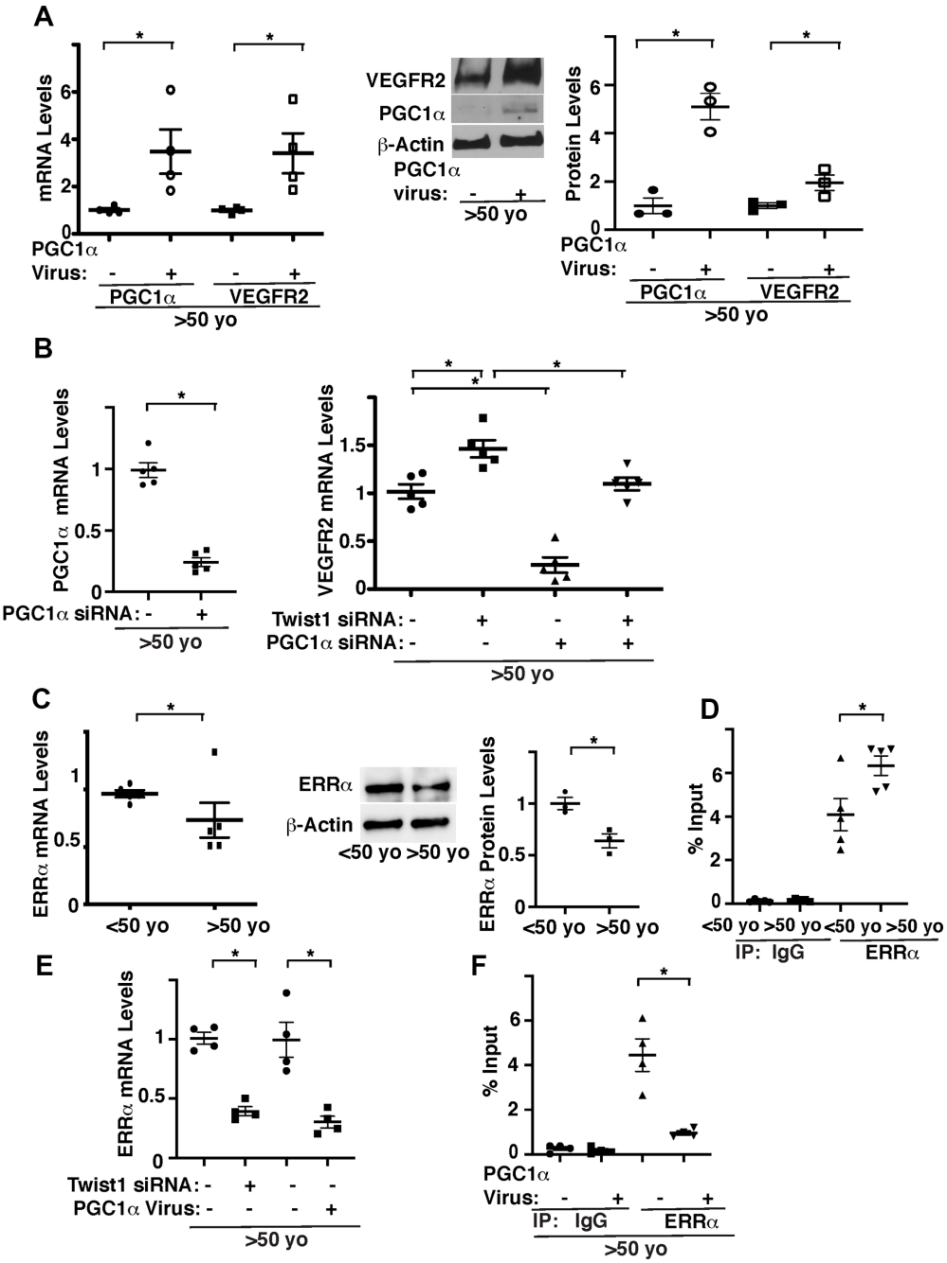
**Twist1 controls age-dependent decline in VEGFR2 expression through PGC1α.** (**A**) Graph showing the mRNA levels of PGC1α and VEGFR2 in aged (>50 years old) human adipose ECs treated with lentivirus overexpressing PGC1α or control virus (vector alone) (*left*, n=4, mean ± s.e.m., *, p<0.05). Representative immunoblots showing VEGFR2, PGC1α, and β-actin protein levels in aged human adipose ECs treated with lentivirus overexpressing PGC1α or control virus (*middle*). Graph showing VEGFR2 and PGC1α protein levels normalized by β-actin protein levels in aged human adipose ECs treated with lentivirus overexpressing PGC1α or control virus (*right*, n=3, mean ± s.e.m., *, p<0.05). (**B**) Graph showing the mRNA levels of PGC1α in aged human adipose ECs treated with PGC1α siRNA or control siRNA with irrelevant sequences (*left,* n=5, mean ± s.e.m., *, p<0.05). Graph showing the mRNA levels of VEGFR2 in aged human adipose ECs treated with Twist1 siRNA, PGC1α siRNA, or in combination (*right*, n=5, mean ± s.e.m., *, p<0.05). (**C**) Graph showing the mRNA levels of ERRα in young vs. aged human adipose ECs (*left*, n=5, mean ± s.e.m., *, p<0.05). Representative immunoblots showing ERRα and β-actin protein levels in young vs. aged human adipose ECs (*middle*). Graph showing ERRα protein levels normalized by β-actin protein levels in young vs. aged human adipose ECs (*right*, n=3, mean ± s.e.m., *, p<0.05). (**D**) ChIP analysis showing the immunoprecipitation levels of VEGFR2 promoter coimmunoprecipitating with control IgG or ERRα antibody in human adipose ECs isolated from young (<50 years old) vs. old (>50 years old) adipose tissues (n=5, mean ± s.e.m., *, p<0.05). (**E**) Graph showing the mRNA levels of ERRα in aged human adipose ECs treated with Twist1 siRNA or PGC1α virus (n=4, mean ± s.e.m., *, p<0.05). (**F**) ChIP analysis showing the immunoprecipitation levels of VEGFR2 promoter coimmunoprecipitating with control IgG or ERRα antibody in human adipose ECs isolated from old (>50 years old) adipose tissues treated with PGC1α virus or control virus (n=4, mean ± s.e.m., *, p<0.05).

We next examined whether Twist1-PGC1α signaling controls VEGFR2 expression. Overexpression of PGC1α using lentiviral transduction, which increases PGC1α mRNA expression by 3.2-times, increased the mRNA and protein levels of VEGFR2 in ECs isolated from aged human adipose tissues (Figure 2A). PGC1α knockdown also inhibited Twist1 knockdown-induced increase in VEGFR2 in aged human adipose ECs (Figure 2B), suggesting that knockdown of Twist1 upregulates VEGFR2 expression through PGC1α in aged ECs.

It is known that PGC1α controls angiogenesis [33–35, 42] by binding to the transcription factor, estrogen-related receptor α (ERRα) [30, 43]. While ERRα stimulates angiogenic factor expression in muscle cells, ERRα represses angiogenic factor expression in ECs [44]. ERRα binds to DNA sites with the consensus sequence TCAAGGTCA (ERR response element (ERREs)) [45, 46]. Since VEGFR2 promoter sequence contains ERRE sites, we examined the effects of aging on ERRα expression and interaction of ERRα and the VEGFR2 promoter region using young vs. aged human adipose ECs. The mRNA and protein levels of ERRα in aged human ECs were 23% and 38% lower, respectively, compared to those in young ECs (Figure 2C). ERRα binds to the VEGFR2 promoter region 1.5-times higher in aged adipose ECs when analyzed using chromatin immunoprecipitation (ChIP) assay (Figure 2D). Twist1 knockdown or PGC1α overexpression decreased ERRα expression (Figure 2E) and PGC1α overexpression decreased ERRα binding ability to VEGFR2 promoter region in aged ECs (Figure 2F). These results are consistent with previous report demonstrating that ERRα acts as a transcriptional repressor in ECs [44] and suggest that overexpression of PGC1α stimulates VEGFR2 transcription by decreasing the binding ability of ERRα to the VEGFR2 promoter region in aged ECs.

### Twist1 and PGC1α mediate age-related decline in angiogenic activities

**Figure 3:**
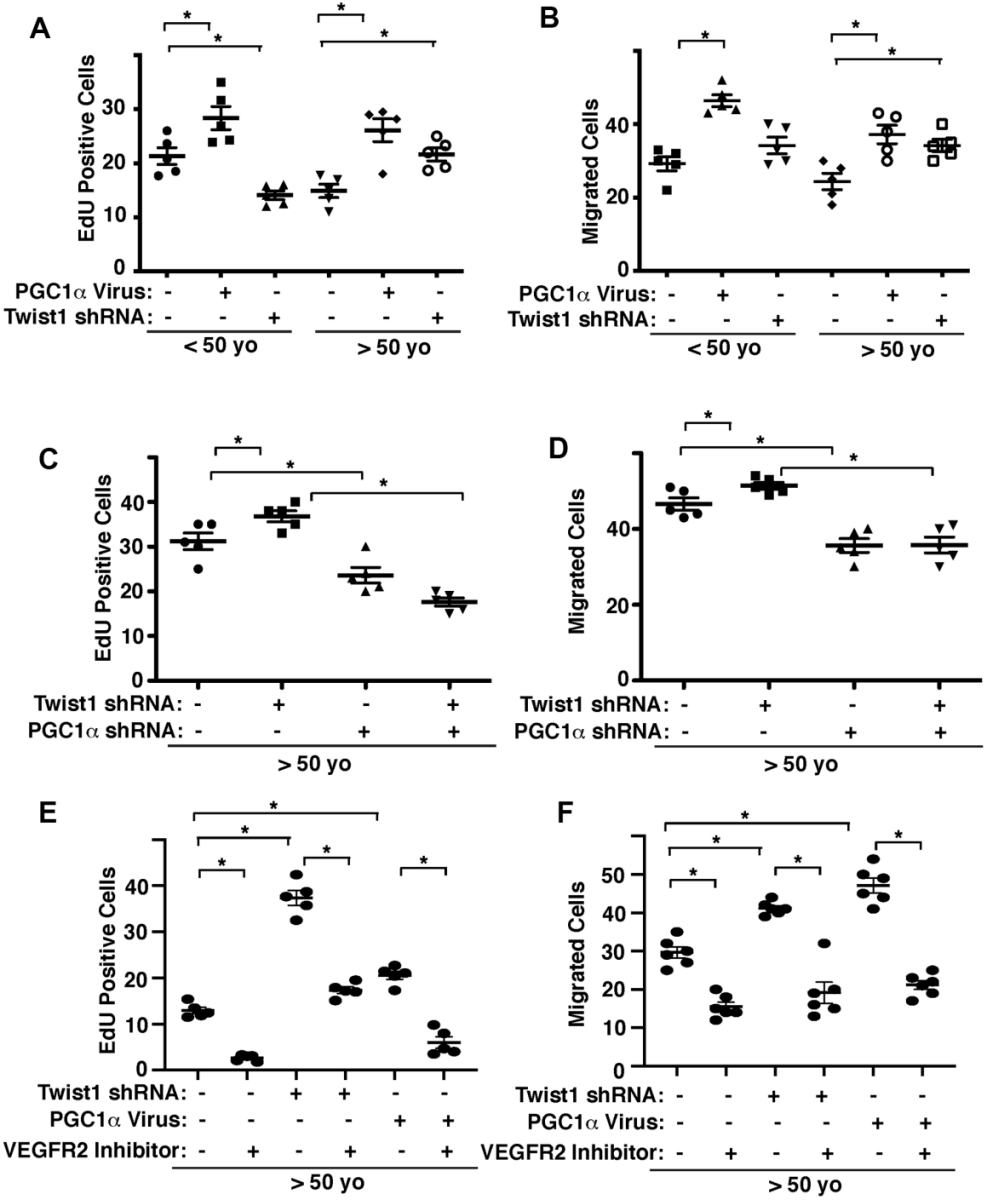
**Twist1-PGC1α signaling controls EC DNA synthesis and migration in young vs. aged ECs.** (**A**) Graph showing EdU-positive young (<50 years old) vs. aged (>50 years old) human adipose ECs treated with lentivirus overexpressing PGC1α or Twist1 shRNA (n=5, mean ± s.e.m., *, p<0.05). As a control, young vs. aged human adipose ECs were treated with lentivirus encoding control shRNA with irrelevant sequences or control virus (vector alone). (**B**) Graph showing young vs. aged human adipose ECs treated with lentivirus overexpressing PGC1α or Twist1 shRNA migrating towards 5% FBS (n=5, mean±s.e.m., *, p<0.05). (**C**) Graph showing EdU-positive aged human adipose ECs treated with lentivirus encoding Twist1 shRNA, PGC1α shRNA, or in combination (n=5, mean ± s.e.m., *, p<0.05). As a control, aged human adipose ECs were treated with lentivirus encoding control shRNA with irrelevant sequences. (**D**) Graph showing aged human adipose ECs treated with lentivirus encoding Twist1 shRNA, PGC1α shRNA, or in combination migrating towards 5% FBS (n=5, mean ± s.e.m., *, p<0.05). (**E**) Graph showing EdU-positive aged human adipose ECs treated with lentivirus encoding Twist1 shRNA, PGC1α, or in combination with VEGFR2 inhibitor SU5416 (n=5, mean ± s.e.m., *, p<0.05). As a control, aged human adipose ECs were treated with lentivirus encoding control shRNA with irrelevant sequences, control virus (vector alone) or control vehicle. (**F**) Graph showing aged human adipose ECs treated with lentivirus encoding Twist1 shRNA, PGC1α, or in combination with VEGFR2 inhibitor SU5416 migrating towards 5% FBS (n=6, mean ± s.e.m., *, p<0.05).

We and other groups have demonstrated that angiogenesis is inhibited in aged ECs [8–12]. We next examined whether Twist1-PGC1α signaling mediates age-related decline in angiogenic activities. When we manipulated the expression of PGC1α or Twist1 in human adipose ECs of different ages using lentiviral transduction, PGC1α overexpression stimulated DNA synthesis and EC migration (Figure 3A, 3B). Twist1 knockdown inhibited DNA synthesis by 41% in young human adipose ECs, whereas it stimulated DNA synthesis and EC migration by 42% and 28%, respectively in aged ECs (Figure 3A, 3B). Twist1 knockdown-induced stimulation of DNA synthesis and migration in aged human adipose ECs was inhibited when treated with PGC1α shRNA in combination (Figure 3C, 3D). Stimulation of DNA synthesis and migration by Twist1 knockdown or PGC1α overexpression in aged human adipose ECs were also inhibited when treated with VEGFR2 inhibitor (SU5416) in combination (Figure 3E, 3F), suggesting that endothelial Twist1-PGC1α signaling mediates age-dependent inhibition of DNA synthesis and migration through VEGFR2.

**Figure 4:**
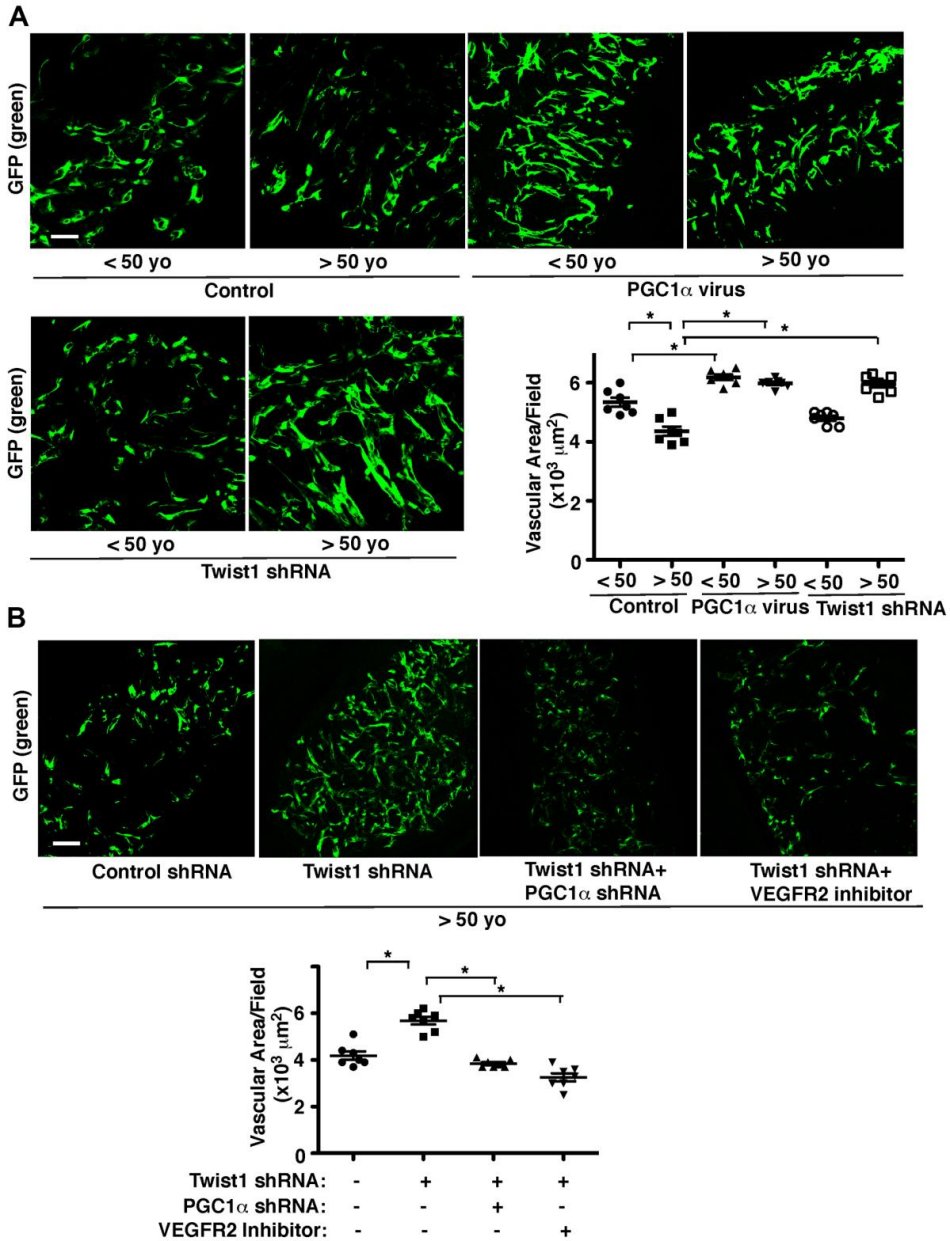
**Twist1-PGC1α signaling mediates age-dependent decline in vascular formation in the subcutaneously implanted gel.** (**A**) IF micrographs of fibrin gel supplemented with GFP-labeled young (<50 years old) vs. aged (>50 years old) human adipose ECs treated with lentivirus overexpressing PGC1α or Twist1 shRNA and subcutaneously implanted on NSG mice for 7 days. As a control, young vs. aged human adipose ECs were treated with lentivirus encoding control shRNA with irrelevant sequences or vector alone. Scale bar, 100 μm. Graph showing vascular area in the gel (n=7, mean ± s.e.m., *, p<0.05). (**B**) IF micrographs of fibrin gel supplemented with GFP-labeled aged human adipose ECs treated with lentivirus encoding Twist1 shRNA or in combination with PGC1α shRNA or VEGFR2 inhibitor (SU5416) and subcutaneously implanted on NSG mice for 7 days. As a control, aged human adipose ECs were treated with lentivirus encoding control shRNA with irrelevant sequences or control vehicle. Scale bar, 100 μm. Graph showing vascular area in the gel (n=7, mean ± s.e.m., *, p<0.05).

To further study age-dependent changes in vascular formation *in vivo*, we used the fibrin gel implantation system, in which young vs. aged human adipose ECs were mixed in the gel [47], and characterized vascular formation in the gel. Consistent with previous report [47], vessel formation derived from supplemented ECs in the gel was attenuated when gel mixed with GFP-labeled aged ECs was implanted under the skin of adult immunocompromised NSG mice. Vascular area was 23% lower than that in the gel supplemented with young ECs (Figure 4A). Blood vessel formation derived from supplemented ECs was stimulated when young and aged human adipose ECs overexpressing PGC1α were supplemented in the gel and subcutaneously implanted (Figure 4A). Twist1 knockdown in young human adipose ECs, which inhibits EC DNA synthesis (Figure 3), tended to inhibit supplemented EC-derived vascular formation (Figure 4A), while Twist1 knockdown in supplemented aged ECs, which increases VEGFR2 expression (Figures 1, 2), restored blood vessel formation in the implanted gel (Figure 4A). Knockdown of PGC1α or VEGFR2 inhibitor suppressed restoration of vascular formation induced by Twist1 knockdown in aged ECs in the gel (Figure 4B), suggesting that Twist1 knockdown restores vascular formation in aged ECs through PGC1α and VEGFR2.

### Endothelial Twist1 mediates age-related decline in post-PNX lung growth

**Figure 5:**
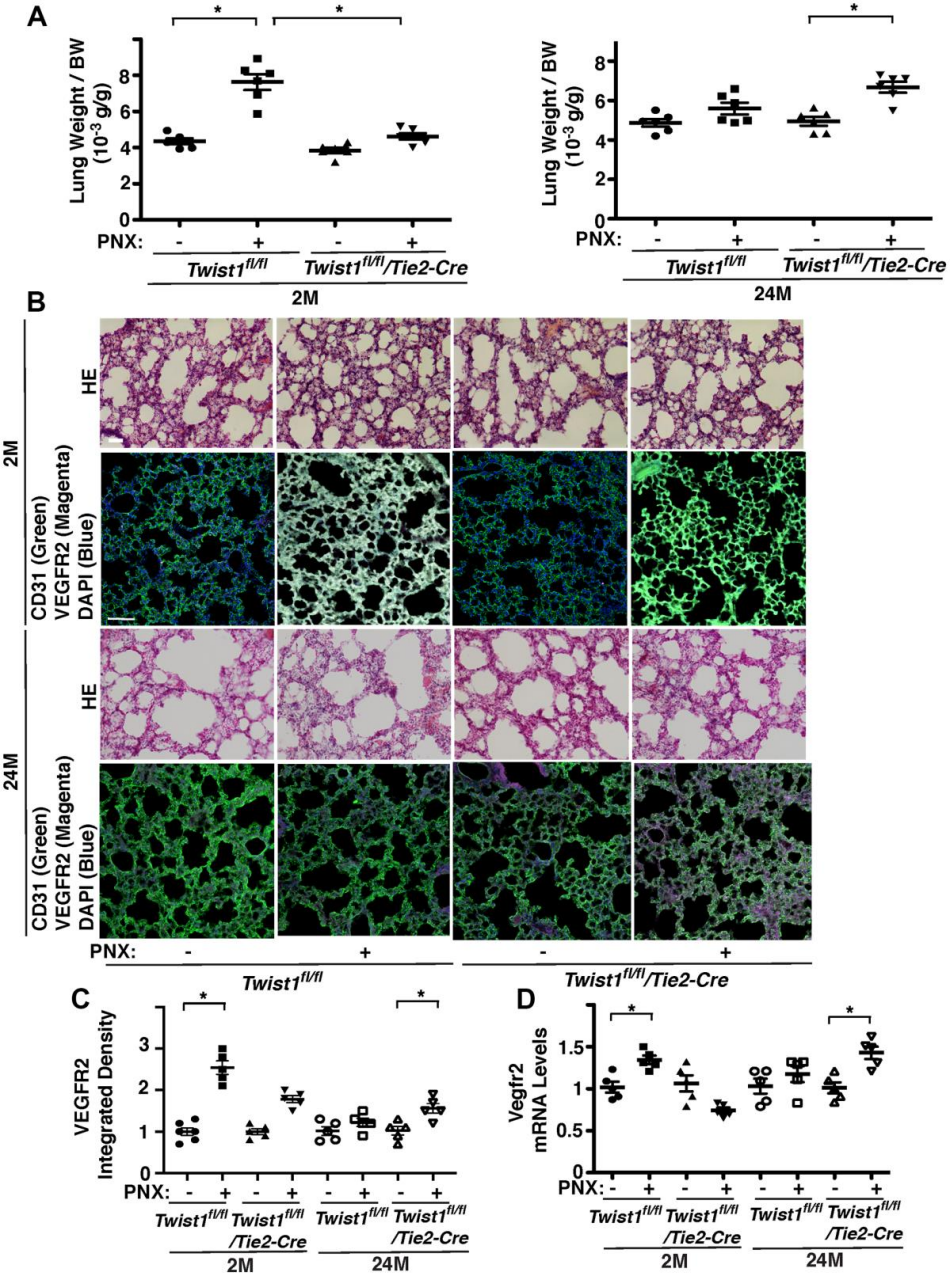
**Endothelial Twist1 mediates age-dependent inhibition of post-PNX compensatory lung growth.** (**A**) Graphs showing the ratio of the weight of right lung cardiac lobe to mouse BW in the 2M vs. 24M old *Twist1^fl/fl^* or *Twist1^fl/fl^*/*Tie2-cre* mice after PNX (n=6, mean ± s.e.m., *, p<0.05). (**B**) H&E-stained mouse lungs in the cardiac lobe of 2M vs. 24M old *Twist1^fl/fl^* or *Twist1^fl/fl^*/*Tie2-cre* mice after PNX (*top, 3^rd^*) Scale bar, 25 μm. IF micrographs showing staining of CD31, VEGFR2 and DAPI in 2M vs. 24M old *Twist1^fl/fl^* or *Twist1^fl/fl^*/*Tie2-cre* mice after PNX (*2^nd^, bottom*). Scale bar, 100 μm. (**C**) Graph showing integrated density of VEGFR2 in 2M vs. 24M old *Twist1^fl/fl^* or *Twist1^fl/fl^*/*Tie2-cre* mice after PNX (n=5-6, mean ± s.e.m., *, p<0.05). (**D**) Graph showing the mRNA levels of Vegfr2 in the ECs isolated from 2M vs. 24M old *Twist1^fl/fl^* or *Twist1^fl/fl^*/*Tie2-cre* mouse lungs after PNX (n=5, mean ± s.e.m., *, p<0.05).

Angiogenesis plays important roles in compensatory lung growth after unilateral PNX, while these effects are inhibited in aged animals [12–14, 48]. Knockdown of Twist1 in aged human adipose ECs reverses age-dependent inhibition of EC DNA synthesis and migration in cultured ECs and blood vessel formation in the gel implantation system (Figures 3, 4). Therefore, we examined the role of endothelial Twist1 in age-dependent inhibition of lung growth after PNX. The ratio of the weight of right cardiac lobe to mouse body weight (BW) increased by 1.8-fold in the 2M old *Twist1^fl/fl^* mouse lungs 7 days after PNX (Figure 5A). The post-PNX lung growth was attenuated in the 24M old *Twist1^fl/fl^* mouse lungs (Figure 5A). The alveolar size measured by mean linear intercept (MLI) decreased by 33% and the number of alveoli increased by 1.7- times in the hematoxylin and eosin (H&E)-stained histological sections of the 2M old *Twist1^fl/fl^* mouse right lung lobe after left PNX (Supplementary Figure 1). These effects were attenuated in 24M old *Twist1^fl/fl^* mouse lungs (Supplementary Figure 1). The mRNA and protein levels of VEGFR2 in the 2M old *Twist1^fl/fl^* mouse lungs 7 days after left PNX also increased by 1.3- and 2.6-times, respectively, while the effects were also attenuated in 24M old post-PNX *Twist1^fl/fl^* mouse lungs (Figure 5B–5D).

To examine the effects of endothelial Twist1 on post-PNX lung growth in the aged lung, we compared lung growth between 2M and 24M old *Twist1^fl/fl^/Tie2-cre* mice, in which Twist1 mRNA levels in mouse lung ECs were 78% lower than those in *Twist1^fl/fl^* mice (Figure 1F). Post-PNX lung growth was inhibited in 2M old *Twist1^fl/fl^/Tie2-cre* mice compared to that in 2M old *Twist1^fl/fl^* mice, while post-PNX lung growth was restored in 24M old *Twist1^fl/fl^/Tie2-cre* mice (Figure 5A). The alveolar size and number were also restored in 24M old *Twist1^fl/fl^/Tie2-cre* mouse lungs after left unilateral PNX (Supplementary Figure 1). The mRNA levels of VEGFR2 did not increase in the 24M old *Twist1^fl/fl^* mouse lung ECs after PNX, while the levels increased by 1.4-times in the 24M old *Twist1^fl/fl^/Tie2-cre* mouse lungs after PNX (Figure 5D). The immunohistochemical analysis also confirmed that the protein levels of VEGFR2 increased by 1.4-times in the post-PNX 24M old *Twist1^fl/fl^/Tie2-cre* mouse lungs (Figure 5C), suggesting that knockdown of endothelial Twist1 increases angiogenic factor expression and restores post-PNX lung growth in aged lungs.

## DISCUSSION

**Figure 6:**
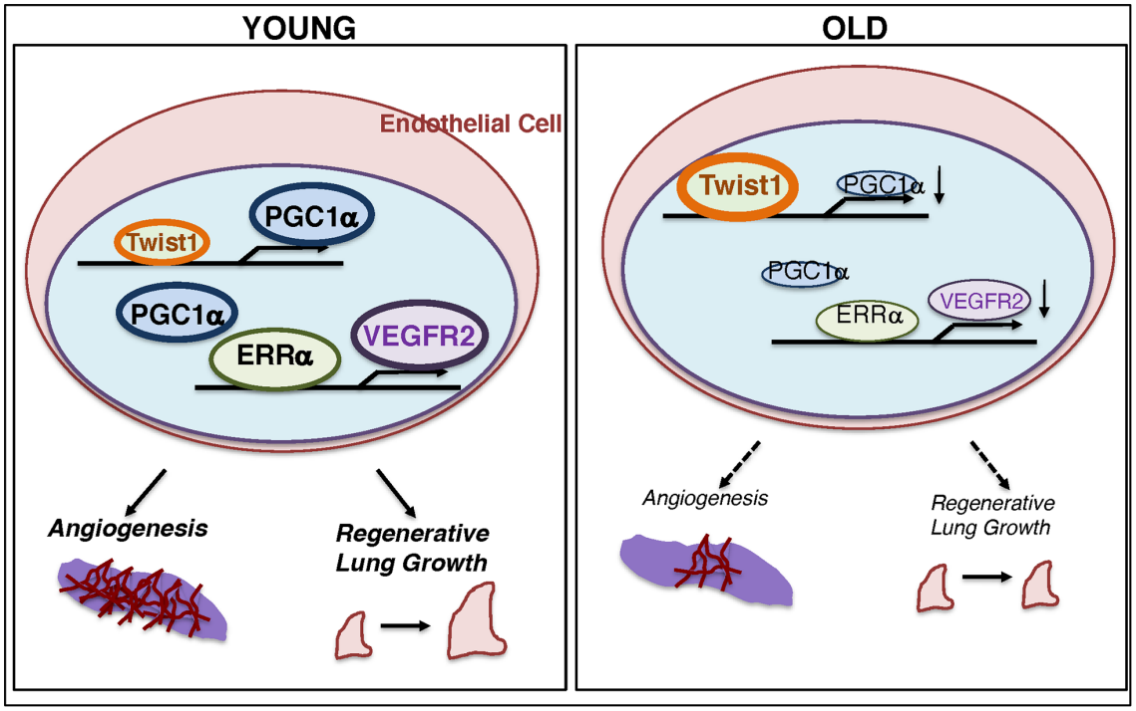
**Schematic illustration of the angiogenic signaling pathways in young vs. aged ECs.** Schematic illustration showing that Twist1 expression is higher in aged ECs, which decreases the expression of PGC1α and VEGFR2, and inhibits EC DNA synthesis and migration *in vitro* and blood vessel formation and alveolar regeneration in aged mice.

Here, we have demonstrated that the levels of Twist1 are higher, while the levels of PGC1α and VEGFR2 are lower in aged human adipose ECs and mouse lung ECs compared to those from young animals. Twist1 knockdown or PGC1α overexpression upregulates VEGFR2 expression in aged ECs, which reverses age-dependent decline in EC proliferation and migration. Vascular formation was suppressed in the fibrin gel mixed with aged ECs, while PGC1α overexpression or Twist1 knockdown in aged ECs restored the effects. Post-PNX lung growth inhibited in 24M old mice was restored in *Twist1^fl/fl^/Tie2-cre* mice. These results suggest that age-dependent upregulation of Twist1 expression in ECs inhibits lung vascular and alveolar morphogenesis in aged mice by decreasing VEGFR2 expression (Figure 6). Modulation of Twist1 expression in ECs could be one of the promising strategies for age-related lung diseases and may be able to delay the aging processes in the lungs.

We examined the mechanism of aging-dependent inhibition of angiogenesis using ECs isolated from human adipose tissues. We then characterized the effects of aging on regenerative lung growth using a PNX model. We used a unilateral PNX model because (1) post-PNX regenerative lung growth takes place after the organs are damaged or partially removed*,* while the process is attenuated in older people [13, 14], (2) angiogenic signaling mediates post-PNX lung growth [12, 49, 50], (3) inhibition of angiogenesis and attenuation of lung regeneration and repair abilities in aged people contribute to the pathogenesis of age-related lung diseases, including COPD [15, 16]. Although lung transplantation is one of the options for end-stage lung diseases, it is not an optimal approach [51–53]. Stimulating the intrinsic ability of lung regeneration may be a promising strategy to restore structures and functions after resection of injured lungs. Thus, although the PNX model does not directly mimic certain diseases, understanding the mechanism of age-dependent inhibition of angiogenesis and regenerative lung growth using this model would improve the strategies for repair from age-related lung diseases.

We found that upregulation of Twist1 mediates age-dependent decline in angiogenesis through PGC1α and VEGFR2 expression. Twist1 controls multiple angiogenic pathways (e.g., angiopoietins (Angs)-Tie2 [18, 25], VEGF-VEGFR2 [24], PDGF [28]). For example, we have reported that Twist1 controls Tie2 expression by binding to its promoter region, E-box [18, 25]. Since the VEGFR2 promoter also contains an E-box, in addition to the pathway through PGC1α-ERRα, Twist1 may control VEGFR2 expression by binding to the E-box promoter sequence. Twist1 also interacts with other pathways controlling angiogenesis (HIF1α [54], Wnt [55], Notch [56, 57], PI3K-AKT [58, 59], and TGF-β [27]). Given that Twist1 binds to PGC1α and inhibits its co-transcriptional activity in fat cells [36], Twist1 may control angiogenesis by changing metabolic signaling. Twist1 is also involved in DNA methylation and cellular senescence, which are associated with aging processes [60–62]. Aged senescent cells secrete a number of senescence-associated secretory phenotype (SASP) factors, which may have broad effects on lung regeneration in aged lungs [63, 64]. Thus, multiple indirect signaling pathways are involved in age-dependent decline in angiogenesis through Twist1. Given that the multiple signaling pathways are required for physiological and functional blood vessel formation [65–67], manipulation of the expression of endothelial Twist1, which is involved in multiple angiogenic signaling mechanisms, could be an optimal strategy to reverse lung vascular and alveolar formation in the aged lung.

Our results have demonstrated that post-PNX lung growth is restored in 24M old *Twist1^fl/fl^/Tie2-cre* mouse lungs. Twist1 knockdown stimulates angiogenic activity in aged ECs and vascular formation in the gel implantation system. Thus, knockdown of Twist1 may stimulate lung growth in aged mice through increasing angiogenesis. In addition to ECs, Twist1 is expressed in other cell types as well (e.g., fibroblasts, epithelial cells) [17, 68], which may also influence lung vascular and alveolar morphogenesis. We used Tie2-specific Twist1 knockout mice in this study and found that Twist1 knockdown in Tie2-expressing cells restores regenerative lung growth in the aged mouse lungs. Since Tie2 is expressed in other cell types such as fibroblasts and immune cells, which also contribute to vascular and alveolar epithelial morphogenesis [69], Twist1 expression in these other cells may contribute to post-PNX lung growth in the aged lung. Twist1 knockdown in ECs in other organs may also indirectly affect post-PNX lung growth. The effects of Twist1 on angiogenesis may be different among ages and tissues. Although knockdown of Twist1 increases VEGFR2 expression in aged ECs and restores vascular and alveolar morphogenesis in aged mice, endothelial Twist1 knockdown decreases VEGFR2 expression and impairs retinal angiogenesis in neonatal mice [24] and inhibits post-PNX lung growth in young mice (Figure 5). Consistently, Twist1 overexpression did not decrease VEGFR2 expression nor inhibit EC proliferation and migration in young ECs; rather Twist1 overexpression increased VEGFR2 expression and stimulated EC DNA synthesis and migration in young ECs (Supplementary Figure 2A–2C). Thus, the effects of Twist1 seem to be age dependent; increased levels of Twist1 in aged ECs decrease PGC1α and VEGFR2 expression and inhibit EC proliferation and migration, while overexpression of Twist1 increases VEGFR2 expression and induces EC proliferation and migration in young ECs. As expected from previous reports using young adipose tissues [36] or skeletal muscles [70], overexpression of Twist1 tended to but did not significantly reduce PGC1α mRNA expression in young ECs (Supplementary Figure 2A). Twist1 is known to behave as a negative regulator of PGC1α in young fat tissues [36], and may indirectly control angiogenesis by changing its activity in young ECs. The levels of Twist1 in aged ECs seem to be high enough to inhibit cell proliferation and migration; Twist1 overexpression in aged ECs did not significantly change DNA synthesis and migration (not shown). Sustained knockdown of endothelial Twist1 in aged *Twist1^fl/fl^/Tie2-cre* mice may modulate other signaling pathways in other cell types and other organs. In fact, knockdown of endothelial Twist1 in aged *Twist1^fl/fl^/Tie2-cre* mice increased VEGFR2 expression but not PGC1α expression (Figure 1D). Further investigation using inducible endothelial-specific Twist1 knockout mice will elucidate the effects of endothelial Twist1 on age-dependent changes in angiogenesis and alveolar regeneration during specific time frames.

We found that binding of ERRα to the VEGFR2 promoter region increased in aged ECs (Figure 2D), in which VEGFR2 expression is suppressed. This is consistent with the previous report demonstrating that ERRα acts as a transcriptional repressor in ECs [44]. However, ERRα expression in aged ECs is lower than that in young ECs (Figure 2C). How aging downregulates ERRα, while increasing ERRα recruitment to the VEGFR2 promoter region remains unclear. Even at the lower expression level, the remaining ERRα recruited to the promoter region may exert its activity. Post-translational modifications of ERRα induced in aged ECs may increase its recruitment to the promoter region. ERRα may also interact with co-repressors and activators on its promoter regions and modulation of these interactions could contribute to regulation of ERRα activity.

In addition to its role in angiogenesis [29, 33–35, 38, 42, 71], PGC1α controls mitochondrial biogenesis [29–32, 42, 71]. PGC1α also regulates a number of anti-reactive oxygen species (ROS) genes [72, 73], which contribute to age-related pathologies, and protects against endothelial dysfunction [29, 74]. Thus, modulation of Twist1-PGC1α signaling may reverse age-dependent impairment of angiogenesis through multiple mechanisms.

Mechanical forces control vascular formation and function [75–78], and appropriate micromechanical environment is necessary for lung development and regeneration [69, 77, 79]. Twist1 is known to mediate the effects of mechanical forces, including shear stress [22], ECM stiffness [80, 81] and stretching forces [82]. Increases in the ratio of collagen and elastin in aged fibroblasts increase pulmonary stiffness and lower compliance [83]. The response to shear stress is also altered during aging [84]. In fact, premature aged ECs from Hutchinson-Gilford progeria syndrome patients impair normal mechanosensing, leading to accelerated fibrosis [84]. We have reported that age-related lung diseases, such as pulmonary fibrosis and accompanied pulmonary hypertension, in which changes in mechanical environment are involved in the disease progression [85–87], are prevented in *Twist1^fl/fl^/Tie2*-*cre* mice [18, 27]. Other transcription factors and co-factors (e.g., TFII-I, GATA2, YAP1) that sense mechanical forces interact with Twist1, control angiogenesis [47, 50, 71, 75, 88], and contribute to age-related lung diseases (*e.g.,* pulmonary fibrosis, pulmonary hypertension) [85–87]. Thus, Twist1 senses age-dependent changes in the mechanical forces to control angiogenesis and lung regeneration.

We used ECs isolated from human adipose tissues of different conditions (e.g., body mass index (BMI), sex, pre-existing diseases, visceral vs. subcutaneous adipose tissues). The heterogeneity of the samples may impact angiogenic signaling. Investigation of the effects of aging on angiogenesis using a more specific cohort with larger sample size will further elucidate the mechanism.

In summary, endothelial Twist1 mediates age-related decline in blood vessel formation and post-PNX compensatory lung growth through PGC1α-VEGFR2 signaling. Modulation of endothelial Twist1 would potentially be a new strategy for aging-associated lung diseases.

## MATERIALS AND METHODS

### Materials

Anti-β-actin (A5316) and –PGC1α (AB3242) monoclonal antibodies were from Sigma (St. Louis, MO). Anti-VEGFR2 (2479) and –ERRα (13826) antibodies were from Cell Signaling (Danvers, MA). Anti-CD31 (102409) and –CD45 (103113) antibodies were from BioLegend (San Diego, CA). Anti-VE-cadherin (562243) and -CD31 (553370) antibodies were from BD Pharmingen (San Diego, CA). Anti-Twist1 antibody (sc-15393) was from Santa Cruze Biotechnology (Dallas, TX). SU5416 was purchased from Millipore Sigma (Burlington, MA).

### EC isolation

Mouse lung ECs were isolated from C57BL6 mice (2M and 24M old) using anti-CD31 conjugated magnetic beads and cultured as reported [27, 28, 50]. De-identified discarded human adipose tissues were collected from Medical College of Wisconsin (MCW) Tissue Bank. These de-identified human adipose tissue-derived ECs have been determined as Non-Human Subjects Research by the MCW Institutional Review Board. Sample demographic information collected using the Generic Clinical Research Database (GCRD) is summarized in Table 1. The samples from cancer patients were excluded. ECs were isolated and cultured as described before [47, 50] and used between passages 1-2.

**Table 1:** Sample demographics.

**Sample demographics (n=16)**	**Young (< 50 y.o., n=8)**	**Old (> 50 y.o., n=8)**
Gender, Male/Female	3 (37.5%)/5 (62.5%)	5 (62.5%)/3 (37.5%)
Age, year (mean ± s.e.m)	34.62±2.96	70.37±2.50
Body mass index (mean ± s.e.m)	28.82±2.91	28.38±1.33
Underlying diseases		
Coronary artery disease	0 (0%)	3 (37.5%)
Hypertension	0 (0%)	3 (37.5%)
Hyperlipidemia	0 (0%)	3 (37.5%)
Diabetes mellitus	0 (0%)	1 (12.5%)
Atrial fibrillation	0 (0%)	3 (37.5%)
None of the above	1 (12.5%)	3 (37.5%)

### Molecular biological and biochemical methods

pLenti-PGC1α (mouse) [89] and pTRIPZ-PGC1α shRNA (human) [90] were obtained as described. Lentiviral construct for human Twist1 shRNA was CCGGGCTGGACTCCAAGATGGCAAGCTCGAGCTTGCCATCTTGGAGTCCAGCTTTTT [28]. pHAGE-Twist1 was constructed as described [27]. The lentiviral pHAGE-GFP construct [75] was used for labeling human adipose ECs. Plasmid with vector only was used as a control. Lentiviral vectors were generated as reported [49, 50, 75]. The siRNA sequences for human PGC1α and Twist1 were described before [18, 25, 27, 28, 71] and siLentFect (BioRad, Hercules, CA) was used for transfection. Transfected human adipose ECs were used for the assays 3 days later. A scrambled siRNA (QIAGEN) was used as a control.

The primers for mouse Twist1, Pgc1α, Vegfr2, and cyclophilin and human Twist1, PGC1α, VEGFR2, and B2M for quantitative reverse transcription (qRT)-PCR were previously described [25, 27, 49, 71, 75]. The primers used for human ERRα were forward; AGGGTTCCTCGAGACAGAG and reverse; TCACAGGATGCCACACCATAG. The iScript reverse transcription kit and iTaq SYBR Green qPCR kit (BioRad, Hercules, CA) were used for qRT-PCR, which was performed using the BioRad real time PCR system (BioRad).

For the chromatin immunoprecipitation (ChIP) assay, DNA from human adipose young vs. aged ECs was immunoprecipitated with the ERRα antibody or control immunoglobulin (Thermo Fisher Scientific, Waltham, MA) [25, 27, 75]. The promoter region of human VEGFR2 binding to ERRα was analyzed with primers, 5’- GTGCCGGTAGGAGAGGATA-3’ and 5’- AGCGGTCAATGTGTGGTC-3’.

### *In vitro* EC DNA synthesis and migration

Human adipose EC DNA synthesis was analyzed by an EdU incorporation assay. Human adipose ECs (EBM2 with 2% serum), in which PGC1α and/or Twist1 expression was manipulated using lentiviral transduction, were treated with EdU (10 μM, 4 h), and analyzed using a confocal Leica SP5 microscope [28]. A modified transwell migration assay was used for analysis of EC migration [28].

### *In vivo* animal experiments

The animal study was conducted following the Guide for the Care and Use of Laboratory Animals of the National Institutes of Health. The Animal Care and Use Committee of MCW reviewed and approved the protocols. Nonobese diabetic/severe combined immunodeficiency gamma (NSG) mice (8 week old; Jackson Laboratory, stock # 005557), C57BL6 mice (Jackson Laboratory, stock # 000664 and NIA/NIH rodent colonies), and *Twist1^fl/fl^/Tie2-cre* and *Twist1^fl/fl^* mice [18, 25, 27, 28] were used for the study. The study used both male and female mice. For gel implantation, we implanted the fibrin gel [27, 28, 47, 50] on the back of NSG mice for 7 days and histological analysis was performed as described [47, 75]. We mixed the gel with SU5416 (final concentration; 3 μM) for VEGFR2 inhibition. Unilateral PNX was conducted as previously described [12, 49, 50]. Histological (MLI, alveolar number) and immunohistochemical analysis was performed as described [12, 49, 50].

### Statistics

All phenotypic analysis was performed by masked observers. Power analysis was conducted to provide 80% power to detect an effective 20-30% difference between the experimental groups. Three or more independent experiments were conducted to determine error bars (SEM) and *p* values. Student’s t-test (two groups) and one-way ANOVA with a post-hoc analysis using the Bonferroni test (more than two groups) were conducted to analyze statistical significance.

## Supplementary Material

Supplementary Figures
