# Supplementary material for: Twist1 signaling in age-dependent decline in angiogenesis and lung regeneration
Source: Aging (Albany NY). 2021 Mar 25;13(6):7781–99. doi: 10.18632/aging.202875 (PMC8034921; doi:10.18632/aging.202875)
Supplement: Supplementary Figures [file aging-13-202875-s001.pdf]

SUPPLEMENTARY FIGURES

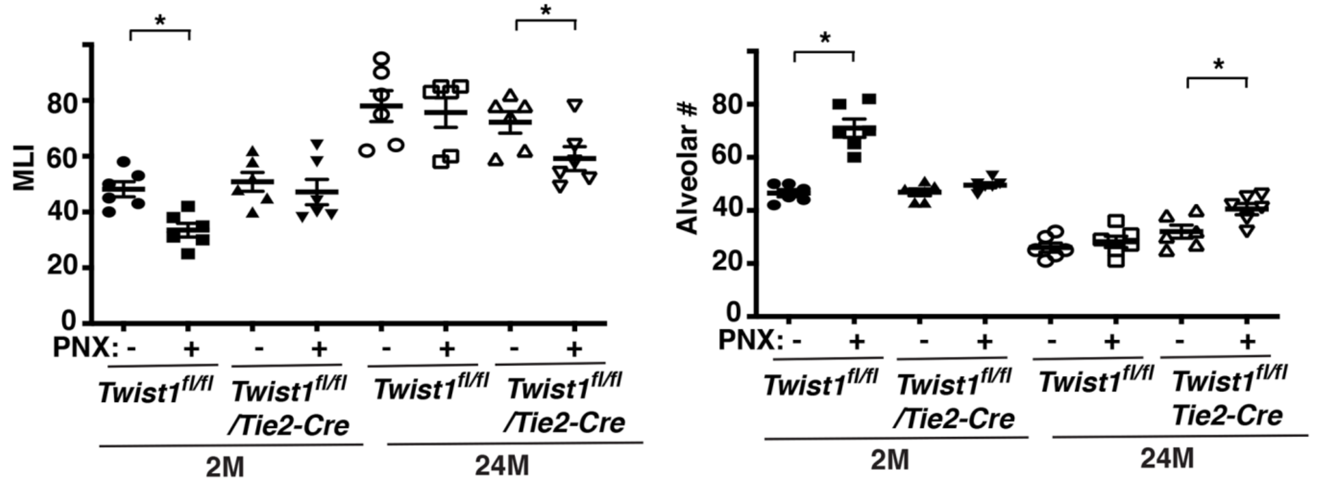

Supplementary Figure 1. Endothelial Twist1 mediates age-dependent inhibition of post-PNX compensatory lung growth. Graphs showing quantification of alveolar size (MLI, *left*) and alveolar number (*right*) in the cardiac lobe of 2M vs. 24M old *Twist1*$^{fl/fl}$ or *Twist1*$^{fl/fl}$*/Tie2-cre* mice after PNX (n=6, mean ± s.e.m., *, p<0.05).

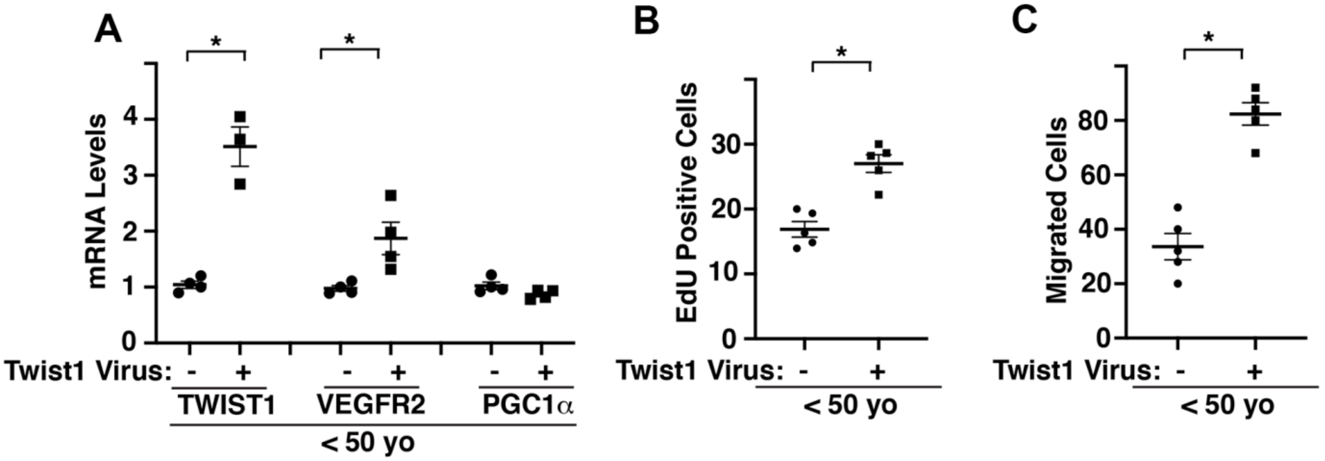

Supplementary Figure 2. Effects of Twist1 on PGC1α and VEGFR2 expression and EC behaviors in young ECs. (**A**) Graph showing the mRNA levels of TWIST1, PGC1α and VEGFR2 in ECs isolated from young (<50 years old) human adipose tissues treated with Twist1 virus or control virus (vector alone) (n=3-4, mean ± s.e.m., *, p<0.05). (**B**) Graph showing EdU-positive young (<50 years old) human adipose ECs treated with lentivirus encoding Twist1 (n=5, mean ± s.e.m., *, p<0.05). As a control, human young adipose ECs were treated with lentivirus encoding control virus (vector alone). (**C**) Graph showing young human adipose ECs treated with lentivirus encoding Twist1 migrating towards 5% FBS (n=5, mean ± s.e.m., *, p<0.05).
